# Theoretical Study of Retinoblastoma in the Hereditary and Non-hereditary Processes Including the Cancer Growth

**DOI:** 10.1007/s11538-025-01483-5

**Published:** 2025-06-26

**Authors:** Hiroshi Toki, Yoshiharu Yonekura, Yuichi Tsunoyama, Masako Bando

**Affiliations:** 1 Osaka University, Research Center for Nuclear Physics (RCNP), Ibaraki, Osaka 567-0047 Japan; 2 Kyoto University, Agency for Health, Safety and Environment, Kyoto, Kyoto 606-8502 Japan; 3 Kyoto University, Yukawa Institute for Theoretical Physics, Kyoto, Kyoto 606-8502 Japan

**Keywords:** Retinoblastoma, Hereditary, Non-hereditary, Mutation, Cancer growth

## Abstract

The two-hit model proposed by Knudson for retinoblastoma has been widely recognized as a standard model for cancer incidence. It successfully predicted the existence of the tumor suppressor gene known as "Rb1" by effectively demonstrating the overall patterns observed in clinical data covering both bilateral and unilateral retinoblastoma cases. However, it is important to note that the model’s prediction currently deviates significantly from clinical data, both qualitatively and quantitatively. Regrettably, this disparity has remained unresolved. In light of this, we conducted a thorough re-evaluation of Knudson’s two-hit model and arrived at a plausible solution that an additional somatic mutation mechanism is required to accurately replicate the magnitude and age dependence observed in both bilateral and unilateral retinoblastoma cases. This revelation offers a fresh and valuable perspective on the development of cancer, highlighting the significance of mutations not only during the cell growth period but also after the retina organ has reached maturity. We refer to this phase as the "mature period," during which the mutation rate has been observed to surpass that of the growth period. With this enhanced understanding of retinoblastoma (Rb), we believe we have shed light on the intricate relationship between somatic and germline mutations. Moreover, this insight provides a promising clue for further exploration into the broader context of cancer incidence resulting from genetic mutations.

## Introduction

The recent advancements in genome sequencing technology have paved the way for the practical implementation of "precision medicine." Utilizing the powerful tool known as Next-Generation Sequencing (NGS), we can now examine genetic abnormalities in cancerous tissues and tailor individualized clinical plans accordingly. Despite the rising expectations to unravel the intricate mechanisms underlying cancer development, particularly regarding the role of mutations and their connection to heredity, the precise quantitative mutation mechanism in somatic cells remains elusive.

From the mid-1900s, scientists who performed intensive animal experiments focused on retinoblastoma (Rb) in terms of genetic effects (Muller 1950; Neel 1947), while immunologists developed the idea of the multi-hit theory (Armitage and Doll 1954). Knudson proposed 2-hit model demonstrating the famous survival functions with only 48 unilateral and bilateral cases and predicted the tumor suppressor gene *Rb1* (Knudson 1971, 2001). This gene was later found as a tumor suppressor gene, and retinoblastoma was confirmed to be initiated from Rb-depleted cone precursors in cultured human retinal cells (Friend et al. 1986; Xu et al. 2014; Singh et al. 2018). The two-hit model, which Knudson proposed by applying the Armitage-Doll’s multi-hit theory (Armitage and Doll 1954) to the simplest Rb, was indeed epoch-making and has been known as an established standard model of cancer incidence. However, in order to apply to the real clinical incidence rates, we have to establish the relationship between the somatic mutation rate and cancer incidence.

Our starting question is "Does this standard two-hit model reproduce the observed clinical data?" Despite its apparent adherence to the data, it is important to note that both the quantity and age distribution significantly deviate from the data (Knudson 1971; Mastrangelo et al. 2008; Schappert-Kimmijser et al. 1966). Notably, the figures for unilateral Rb data exhibit significant deviations of magnitudes unless one takes one-order larger somatic mutation rate. The predicted incidence rate for non-hereditary cases falls approximately one order of magnitude below the observed incidence. The mutation arises during retinal cell development prior to birth, and a time lag before the manifestation of retinoblastoma is expected. This time lag was not considered in the comparison with the clinical data. Unfortunately, the quantitative and qualitative consistency has since gone unchecked. We have re-examined the model and found that the data requires *two different periods of mutation*, while Knudson only considered mutations during the cell growth period.

We here propose 2-period 2-hit model (2P2H), where the term, *period*, is used instead of stage to distinguish from the cancer stage in the clinical term. Our model provides a new perspective on the mechanism of cancer development, showing the importance of mutations not only during cell growth but also in the mature period. This opens up new avenues for cancer research.

Retina consists of normal, 1-mutated and 2-mutated cells, denoted as Rb$^{+/+}$, Rb$^{+/-}$ and Rb$^{-/-}$, with the cell numbers $N_0, N_1$ and $N_2$, respectively. In the hereditary (H) group, every fetus starts with one zygote cell of Rb$^{+/-}$, while it is Rb$^{+/+}$ in the non-hereditary (N) group. The former belongs to H group, and the latter N group. These fertilized eggs differentiate very shortly yielding retinal stem cells (RSC). Thus, they can be classified into 2 groups at the individual level, H and N groups with RSC=Rb$^{+/-}$ and RSC=Rb$^{+/+}$, respectively. The probability of germline mutation to cause Rb$^{+/-}$ is known as $3\times 10^{-5}$/generation (Johnsson et al. 2017). It is important to highlight that the order of magnitude of $10^{-5}$ per locus, which represents the germline mutation frequency, is consistently observed across various animal species with different lifespans (Gondo 2019). Henceforth, we shall refer to this value as the "magic number."

**Fig. 1:**
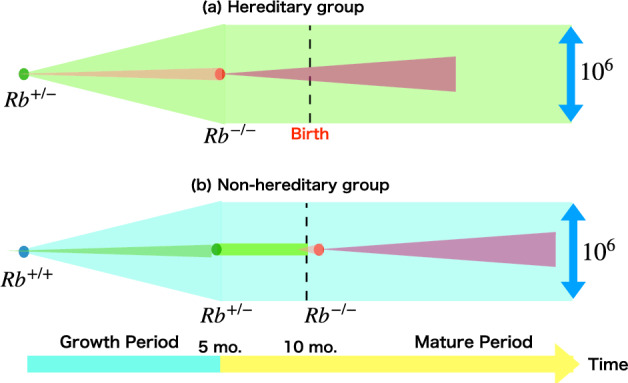
An illustration of mutations in the Rb1 gene in retinal cells and the proliferation of cancer cells is presented for the hereditary group (a) and the non-hereditary group (b). In the hereditary group, the initial zygote cell is Rb$^{+/-}$, shown in green, and during the growth period it expands to approximately $10^6$ cells, with nearly one cell transitioning to Rb$^{-/-}$, shown in red. This double-mutated cell undergoes clonal expansion, leading to cancer, as indicated by the expanding violet zone. In contrast, in the non-hereditary group, the initial zygote cell is Rb$^{+/+}$, shown in blue, and during the growth period it expands to approximately $10^6$ cells, with one cell becoming Rb$^{+/-}$. This cell does not proliferate further until a second mutation occurs. We hypothesize that the second mutation must occur shortly after birth, resulting in an Rb$^{-/-}$ cell, which then undergoes clonal expansion to develop into retinoblastoma, as indicated by the expanding violet zone

It is crucial to emphasize that not only Knudson but also most of the scientists involved in the multi-hit theory have overlooked an important aspect. Ever since Madame Curie proposed the hit theory assuming the sensitive zone, which centered around the existence of susceptible regions, the application of the hit theory has primarily been focused on phenomena occurring at the cellular level (Curie 1929). However, it is essential to recognize that this mutation profile at the cellular level should be regarded as a distinct stage, separate from the stage of the clinical incidence, especially in estimating childhood cancer case. There should be a duration between the occurrence of a cell mutation and the diagnosis of a tumor at the *individual level*, the importance of which has been discussed by many authors (Ruehm et al. 2017; Little 2010). Moreover, in the case of the retina, clinical evidence is observed after birth, while cellular mutations occur even before birth, as illustrated by the expanding violet-colored area in Fig. 1. We will describe in detail the mechanism of cancer cell formation in both the H and N groups in the formulation section.

## Theoretical Framework

First, let us look at how the Knudson scenario affects the absolute values and age distribution in clinical data. In this case, we need to consider the time lag between the occurrence of the mutation at the cellular level and the actual diagnosis of retinoblastoma, otherwise it will not lead to real clinical data. This effect was not considered in the Knudson paper, but it is a process that should be included. Therefore, we will show the results by incorporating the effect of this time lag by introducing a latent function, which is now commonly incorporated.

### Hereditary Group

We begin with the bilateral Rb in the H group, the illustration of which is shown in Fig. 1(a). Noting that the key figure (magic number) is close to the total incidence of bilateral Rb per infant, Knudson correctly predicted that almost all infants in the H group would get a second hit during the growth period (Knudson 1971). The retinal organ (approximately $10^6$ cells) is nearly completed in the fetal period (up to 10 months). Assuming that approximately one cell receives a second hit during this growth phase (Knudson 1971), we proceed to quantify this scenario using differential equations with respect to time.

We consider the case in which mutations occur during the growth period of retinal cells.  We formulate coupled differential equations for the numbers $N_0$, $N_1$, and $N_2$, corresponding to Rb$^{+/+}$, Rb$^{+/-}$, and Rb$^{-/-}$ retinal cells, respectively.  The proliferation rate $\lambda$ and the mutation rate *a* govern how retinal cells grow and acquire mutations. We emphasize here that the mutation rates for the first and second hits are assumed to be the same. We also introduce the maximum number of cells, $N_m$, beyond which proliferation ceases. 

1 $$\begin{aligned} &  \frac{d N_0}{dt}=(\lambda - a)N_0\left( 1-\frac{N_0+N_1+N_2}{N_m}\right) ~, \end{aligned}$$

2 $$\begin{aligned} &  \frac{d N_1}{dt}=((\lambda - a)N_1+aN_0)\left( 1-\frac{N_0+N_1+N_2}{N_m}\right) ~, \end{aligned}$$

3 $$\begin{aligned} &  \frac{d N_2}{dt}=(\lambda N_2+aN_1)\left( 1-\frac{N_0+N_1+N_2}{N_m}\right) ~. \end{aligned}$$

**Fig. 2:**
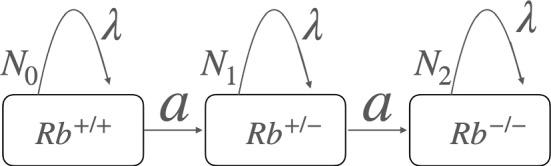
The coupled differential equations (1), (2), (3) for the numbers of cells, $N_0$, $N_1$, and $N_2$, corresponding to Rb$^{+/+}$, Rb$^{+/-}$, and Rb$^{-/ -}$, respectively, are illustrated. Here, $\lambda$ denotes the proliferation rate, and *a* denotes the mutation rate. The maximum number of cells is given by $N_m$. To reproduce the observed frequency of unilateral retinoblastoma, Knudson’s paper required increasing the mutation rate for the second hit by an order of magnitude (Knudson 1971)

We illustrate these coupled differential equations in Fig. 2.  The three stages of retinal cells are represented by squares, corresponding to Rb$^{+/+}$, Rb$^{+/-}$, and Rb$^{-/-}$.  These cells proliferate at a rate $\lambda$, with the maximum number of cells given by $N_m$.  During proliferation, mutations occur at a mutation rate *a*.  Since there are only three stages, the mutation term from $N_2$ does not appear in Eq. (3).  The relationship between these coupled differential equations and the stochastic equations is discussed in Appendix A. These differential equations demonstrate that mutations occur during the course of cell proliferation.

**Fig. 3:**
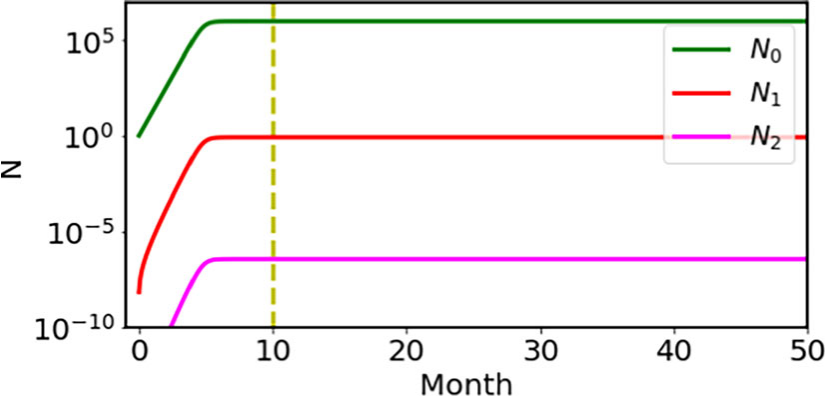
The numbers of cells during the growth period. In the hereditary case, the number of Rb$^{+/-}$ cells increases to $10^6$, as shown by the green curve, while the number of Rb$^{-/-}$ cells reaches nearly 1, as shown by the red curve. In the non-hereditary case, the number of Rb$^{+/+}$ cells grows to $10^6$ shown in the green curve, the number of Rb$^{+/-}$ cells reaches nearly 1, and the number of Rb$^{-/-}$ cells reaches approximately $10^{-6}$

We solved the coupled differential equations numerically. We set the parameters as the maximum number $N_m=10^6$ (Knudson 1971), the growth rate $\lambda =0.1$ /day and the mutation rate $a=6.8\times 10^{-9}/$day. We show the calculated results in Fig. 3 for the second mutation in the hereditary group, and the first and second mutations in the non-hereditary group. The mutations take place within about 150 days (5 months) in the growth period in the fetus time before birth, and the numbers of cells do not increase since then as shown by the flat curves. In this parameter choice, the value for the first hit is 0.89, which provides the ratio of the unilateral and bilateral Rb becomes 1/4 in the H group, where the probability for the bilateral Rb should be $0.89^2=0.79$, and that for the unilateral Rb should be $2\times 0.89\times (1-0.89)=0.20$. The mutation frequency per locus per mitosis is obtained as $r=a\log _e 2/\lambda =4.7\times 10^{-8}$.

With the above values of the parameters, it turned out that the ratio of bilateral and unilateral Rb was 1/4 in the H group. The main process leading to bilateral Rb in the H group is shown schematically in Fig. 1a, where the second mutation makes 0.89 cell out of $10^6$ Rb$^{+/-}$ retina cells to be Rb$^{-/-}$ illustrated by red color, which starts to grow by cloning to develop cancer in retina, which is indicated by the increasing violet zone. Most of Rb cells remain $R^{+/-}$, which is illustrated by the green color in Fig. 1a. To compare with clinical incidence, it is necessary to formulate the clonal proliferation process of cancer and the history of diagnosis. In particular, the cases in the H group where the second mutation occurs before birth, the time lag from the mutation to diagnosis must be taken into account. This will be discussed further below.

The Latent Effect

To bridge the gap between the cellular and individual levels, we introduce the latent effect using a log-normal function, which links the time profile of the number of mutated cells to the clinical incidence rate (Day and Walter 1984). We should introduce the latent effect to take into account the time for the cancer to grow by cloning from one cell at the cell level to the individual level to be recognized as a cancer tissue in order to calculate the clinical incidence rate *I*. We take the convolution integral of the mutation rate $\bar{N}$ by the latent function *f* for the cancer to grow.

4 $$\begin{aligned} I(t)=\int _0^t ds \bar{N}(s) f(t-s) \end{aligned}$$

where $\bar{N}$ is the formation rate of cancer cell at the cell level and the function *f*(*t*) is the latent function expressed by the log-normal function, which is written as

5 $$\begin{aligned} f(t)=\frac{1}{\sqrt{2\pi }\sigma t}\exp \left( -\frac{(\log t-\log \mu )^2}{2 \sigma ^2}\right) \end{aligned}$$

Here, $\mu$ roughly represents the time required for a cancer cell to develop into a clinically detectable cancer at the individual level.

**Fig. 4:**
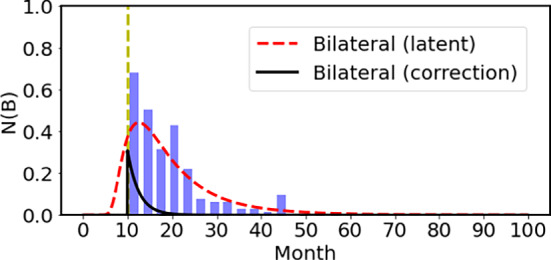
The bilateral Rb using the mutation rate in the growth period and the latent effect for cancer to grow. The results after taking into account the latent effect are shown by the red dashed curve. The contribution from the patient before the birth is not observed clinically, and this amount is redistributed using an exponential function with the width of 2.5 months after the birth, which is shown by the black solid curve

Prenatal Onset, Postnatal Diagnosis

We describe first the latent time effect to connect the mutation rate and the clinical incidence rate for the bilateral case. In order to compare with the clinical incidence rate, which is the number of patients diagnosed by doctors after the birth, we have to shift the cancer patients before the birth to those after the birth. We did this correction using the following expressions.

6 $$\begin{aligned} I(t)=I_0 b\exp (-bt)~, \end{aligned}$$

where $I_0$ is the total number of patients before the birth indicated by the dashed curve in Fig. 4, and this value $I_0$ is known as the area below the dashed curve. Using $b=0.4/$month, we got the clinical incidence after the birth shown by the black curve.

**Fig. 5:**
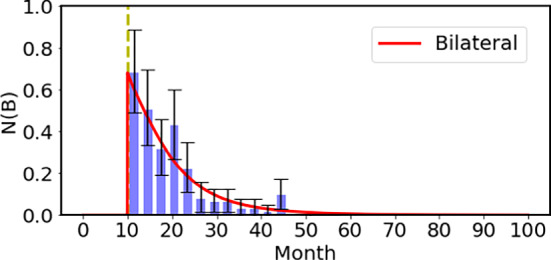
The bilateral Rb using the mutation rate in the growth period and the latent effect for cancer to grow. The vertical axis denotes the number of patients per three months for 100,000 infants. After taking into account the time lag effect and the redistribution of the patients before the birth to after the birth as shown in Fig. 4, we obtained the net result shown by the solid red curve, which is compared with the observed data with Poisson error bars (Mastrangelo et al. 2008)

First of all, the mutation frequency of the *Rb1* gene in the hereditary group has to be fixed. We take the mutation frequency as $3\times 10^{-5}$/generation (Johnsson et al. 2017). We fix the mutation rate *a* in Eq. 2 so that the total incidence of bilateral Rb is $2.4 \times 10^{-5}$ per infant, which corresponds to the observed value. Here, we fixed the parameters of the latent function as $\sigma ^2=0.44$ with the 95% confidence interval of (0.32, 0.73) and $\mu$=11.8 month with the 95% confidence interval of (10.6, 13.2) so as to reproduce the age profile of the bilateral Rb using the maximum likelihood method. The 95% confidence interval was determined using the profile likelihood method, based on the threshold of $\Delta \log L = 2$. The resulting incidence rate of the bilateral Rb are compared nicely with the observed clinical data of Mastrangelo and his collaborators (Mastrangelo et al. 2008) as shown in Fig. 5. The exponential clinical incidence of bilateral Rb reflects both latency and the shift from prenatal onset to postnatal diagnosis. Although comparison is made here with the 378 data points of Mastrangelo et al., the bilateral cases of the 48 data points of Knudson are consistent with the theoretical results.

Again we should stress that the method to reproduce the clinical incidence rate is totally different from what has long been treated in the multi-hit theory as discussed in Appendix B (Armitage and Doll 1954). Having all the above discussions in mind, let us proceed to make analysis of the unilateral case.

### Non-hereditary Group

**Fig. 6:**
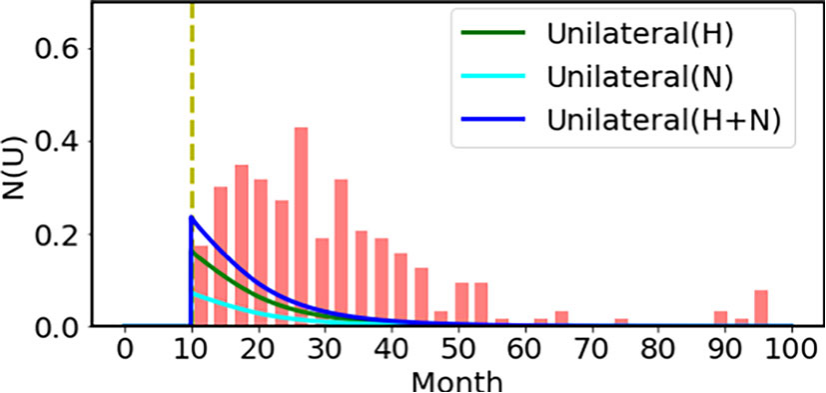
Shown here is the calculated results using only the mutation process in the growth period in the H group (green curve) and additionally the contribution form the N group (cyan curve). We use the same mutation mechanism leading to the first and second hits in the growth period in the N group. The mutation rates are shown in Fig. 3 in the growth period, using which the latent effect was taken into account to get the results. The net incidence rate is shown in blue curve to be compared with the observed clinical data

As noted above, in the H group, not all the infant becomes bilateral Rb, and about 1/4 of the hereditary infant turns out to become unilateral Rb. This was already pointed out by Knudson (2001). As for the N group, during the growth period, about one mutated cell (Rb$^{+/-}$) is expected out of $10^6$ (Rb$^{+/+}$) retina cells just in the same manner in the growth period as seen in the H group. This means the second mutation can occur with about $10^{-6}$ probability in the growth period. Here let us notice the important fact that the observed clinical incidence of unilateral cases is of the order, $3\times 10^{-5}$, and the profile of the clinical incidence rate after the birth is quite different from the 2-hit model prediction in the growth period. Indeed, by taking account the latent effect, the calculated incidence rate in the N group is shown by cyan curve, which is far from the observed results as shown in Fig. 6. Here, we include the contribution of the H group on the unilateral case (green curve), which is about 1/4 of the bilateral case. The total incidence rates are shown by the blue curve. Although the two hits are considered in the growth period, the magnitude and the incidence profile of the unilateral cases are not at all reproduced. We emphasize that the incidence profile of unilateral cases cannot be reproduced if the mutation occurs during the growth period, even when the mutation rate is increased by an order of magnitude (Knudson 1971).

The calculated result of incidence rate for the unilateral case is far from the observation, if we continue the Knudson scenario. This discrepancy demands that the second mutation should occur after the cell proliferation has been terminated, which we name the mature period. Knudson was surely aware of the fact that approximately 60% of all cases are unilateral (B: U = 40%: 60%) most of which do not come from the H group. However, he made neither clear numerical estimation nor made explanation of why its incidence probability was the same order ($10^{-5}$: magic number) as the bilateral cases. Apparently, the data of the U case demands another hit mechanism during the M period. This serious discrepancy could not have been noticed so far as we examine only the normalized survival function (Knudson 1971).

As a preliminary trial, we introduce a simplified mutation process without proliferation, while retaining the mutation rate used during the G period. The methodology and results are presented in Appendix C. This approach, however, fails to reproduce the observed incidence rate of unilateral cases. We found that there are two serious flaws in the maturational mutation scenario with the same mutation rate, which demands two amendments. One is to increase the mutation rate so that the peak value of the incidence rates are reproduced, and the second is to introduce a mechanism to reduce the over production of the clinical data after 5 years. We mention here that Tomasetti et al. introduced mutation in the renewal phase after the cell proliferation period for cancer formation (Tomasetti et al. 2013). The same scenario was proposed by Moolgavkar and Knudson (1981) under the name of two stage model. In our setting, we claim that mutation takes place by another mechanism in the mature period when organs get started to operate their function.

### 2P2H Model

Here, we propose the two-period two-hit (2P2H) model based on the previous discussions. First, we have shown that mutations occurring during the growth period alone cannot account for the incidence rate of unilateral Rb. Therefore, it is necessary to introduce an additional mutation mechanism operating during the mature period. When we assumed that the second hit in the mature period simply reflects a mutation without removal, we were unable to reproduce the incidence rate of unilateral Rb, particularly at later ages. Thus, it became essential to suppress the overproduction of mutated cells. For the mature (M) period, we consider the duration after birth, when the Rb cone tissue begins to function.

**Fig. 7:**
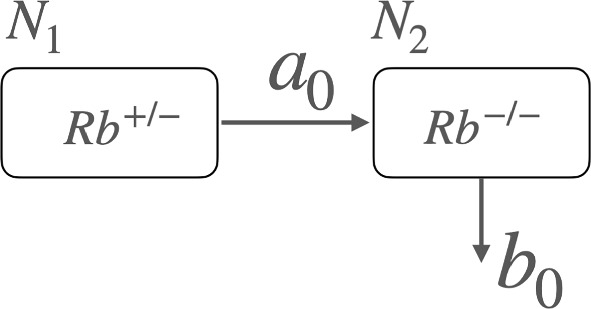
An illustration of the WAM differential equation. The number of semi-mutated cells, $N_1$ ($\textrm{Rb}^{+/-}$), decreases at a mutation rate $a_0$, leading to an increase in the number of fully mutated cells, $N_2$ ($\textrm{Rb}^{-/-}$), which are subsequently reduced at a removal rate $b_0$

We propose to express the mutation dynamics during the mature period using the following equations.

7 $$\begin{aligned} &  \frac{d N_1(t)}{dt}=-a_0 N_1(t)~,\nonumber \\ &  \frac{d N_2(t)}{dt}=a_0 N_1(t)- b_0 N_2(t)~. \end{aligned}$$

This formulation was introduced in the mutation model known as the WAM model, which was designed to account for the observation that various animals exhibit approximately the same mutation rate per generation (Bando et al. 2019; Muller 1927; Russell 1951). We illustrate the WAM model differential equation in Fig. 7. To reproduce the age profile of the incidence rate in the postnatal period, as discussed above, it is necessary to increase the mutation rate $a_0$, particularly around the time of birth. Here, we also consider mechanisms to enhance the mutation rate while compensating for its potentially large value by introducing the removal effect with the rate $b_0$.

**Fig. 8:**
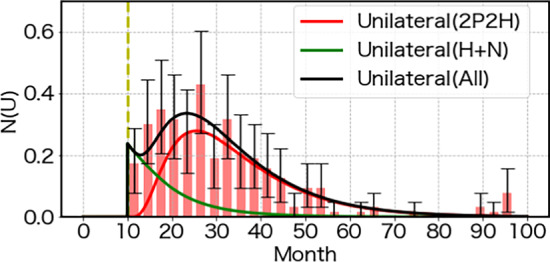
The unilateral incidence rate is compared with the 2P2H model as function of time in unit of month. The contribution from the mutation in the mature period is shown by the red curve and that in the growth period by the green curve. The net result is shown by the black curve, which compare well with the clinical incidence rates with the Poisson error bars (Mastrangelo et al. 2008)

We are left now with the mutation rate $a_0$ and the exclusion rate $b_0$ to be fixed from the magnitude as well as the age profile of the unilateral case. We obtain the values of $a_0, b_0$ using the maximum likelihood method, maintaining the latent function fixed by the bilateral case. The role of the $b_0$ term is to limit the duration of mutation so that the incidence rate can be confined within a certain age range.

The results are shown in Fig. 8, where the theoretical prediction curve agrees well with the observed clinical incidence rate. We obtained $a_0 = 2.4 \times 10^{-8}$ with a 95% confidence interval of $(2.0 \times 10^{-8},\ 2.8 \times 10^{-8})$, and $b_0 = 3.3 \times 10^{-3}$ with a 95% confidence interval of $(2.9 \times 10^{-3},\ 3.7 \times 10^{-3})$. The 95% confidence intervals were determined using the profile likelihood method, based on a threshold of $\Delta \log L = 2$. The ratio $F_0=a_0/b_0=0.73\times 10^{-5}$ provides the total amount of mutation due to the 2P2H model in the mature period. Hence, together with the mutation rate in the growth period of 0.89, we find the total incidence of the unilateral case becomes $2.6\times 10^{-5}$. Again we find the same Magic Number in the form of $F_0$, appearing as the so-called steady state condition of endogenous mutation frequency of various animals (Bando et al. 2019; Muller 1927).

**Table 1:** The ratios of bilateral and unilateral Rb in the H and N groups. Experimental data are given by Schappert-Kimmijser et al. (1966), Knudson (1971) and Mastrangelo et al. (2008). Since Knudson and Mastrangelo et al. do not provide information on the separation of the H and N cases, the total is shown under the Unilateral (N) category. These ratios are compared with the predictions of the 2P2H model, where the total incidence of Rb cancer is $5.9\times 10^{-5}$

Rb	Schappert-Kimmijser et al. (1966)	Knudson (1971)	Mastrangelo et al. (2008)	2P2H theory
Bilateral (H)	25-30%	48 ± 30 %	44 ± 6%	40%
Unilateral (H)	10-15%	(0%)	(0%)	10%
Unilateral (N)	55-65%	(52 ± 32)%	(56 ± 7)%	50%

As for the unilateral Rb, mainly the N group is responsible, where the first hit is caused in the growth period, and the second hit is caused in the mature period. Together with a small contribution from the H group to the unilateral Rb, we list all the ratios of the net incidences for the bilateral and unilateral Rb caused by the H and N groups in Table 1. The total incidence for Rb is $5.9\times 10^{-5}$ per infant. Experimental informations are given by Schappert-Kimmijser et al. (1966) , Knudson (1971) and Mastrangelo et al. (2008). Since there was no identification of the H and N groups in the papers of Knudson and Mastrangelo et al., we show only the total amount in the Unilateral (N) slot in the table. These ratios are to be compared with the results of the 2P2H theory, which shows reasonable agreement between the theory and experiment.

## Discussion

Cancer is commonly believed to be driven by mutations in DNA; however, it is also recognized that epigenetic effects occurring in the cellular environment may impact cancer development. In this paper, we investigate the quantitative relationship between the incidence rate of unilateral Rb and a second non-hereditary mutation that occurs during the growth period. While the literature suggests that epigenetic effects contribute to approximately 10% of cancer cases according to Volgenstein, this study simplifies the analysis by assuming a negligible contribution. Nevertheless, if the contribution were found approximately 10%, adjusting the value of parameter $a_0$ by approximately 10% would account for this effect.

## Conclusion

In conclusion, we started out with the Knudson model on the bilateral Rb and then the unilateral Rb. For the hereditary case, about one mutation occurred out of $10^6$ cells in the growth period, and applying the latent effect we were able to reproduce the clinical incidence rates. As for the unilateral Rb of the non-hereditary origin, we found that the mutations in the growth period was unable to provide the clinical incidence rate and demanded another mutation mechanism in the mature period. We proposed a plausible mutation mechanism occurring at the mature stage with a higher mutation rate than that in the growth period, along with a removal effect modeled after the WAM framework. It turned out that the 2P2H theory succeeded in describing all the existing data of the bilateral and unilateral Rb quantitatively. Retinoblastoma, which involves only a single tumor suppressor *Rb1*, clearly taught us that there were two important periods for mutation and the two mutation mechanisms were necessary in reproducing both the unilateral and bilateral cases. Our finding will bridge the long mysterious gap between somatic and germline mutation rates.

The mechanisms of cancer have long been investigated from the scope of cell level with the multi-stage or multi-hit picture (Armitage and Doll 1954). We believe that our model has established a standard approach to formulation focusing on the simplest target such as Rb cancer. The picture of the multi-stage pathway of Rühm et al. showing a time-tracking diagram well illustrates this approach by using the terminology "stage" or "step" changing from normal cells to cancer cells over time (Ruehm et al. 2017). In our case, we use the term "period" to describe the mutation from healthy cells to malignant cells in the diagram, followed by a cancer diagnosis, which pathway is also discriminated by Rühm et al. by introducing the time, $t_{lag}$, until malignant cell progresses towards a clinically manifesting tumor. On the other hand, we proposed to introduce the latent process to bridge between obviously different levels as cell and individual levels. We believe that our proposal opens a door to further development towards the clarification of the basic direction.

## Figures and Tables

**Fig. 9:**
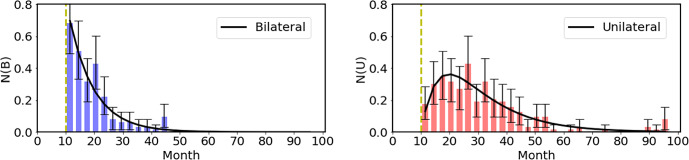
Clinical incidence rates for bilateral (blue) and unilateral (red) cases modeled using the two-hit framework. For the bilateral case, the hit rate is estimated as $k_B = 0.112/\text {month}$, while for the unilateral case, it is estimated as $k_U = 0.071/\text {month}$

**Fig. 10:**
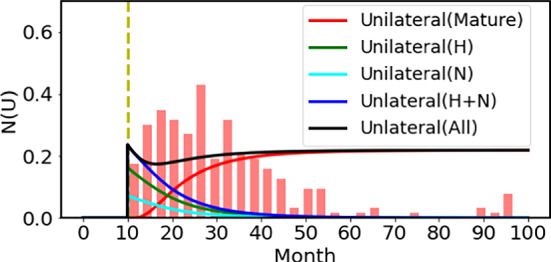
Shown here is the incidence rates including the mutation process in the mature period with the same mutation rate as in the growth period for the unilateral case. The contributions from the growth period both in the H and N groups are shown by the blue curve, and the contribution from the mature period in the N group by the red curve. The net result is shown by the black curve to be compared with the observed incidence rate shown by the red vertical bars (Mastrangelo et al. 2008)

## Appendix A: The Relationship Between Probability Models and Differential Equations in Proliferating Cell Dynamics

The mutation of bacteria from virus sensitivity to virus resistance was studied by Lulias and Delbrück (1943). They employed differential equations to describe bacterial growth and mutation processes, similar to those used in this paper for modeling cell proliferation and mutations. A general differential equation framework was later adopted by Sachs and Hlatky (2010). On the other hand, stochastic models have been employed by several authors to address similar problems, such as those investigated by Iwasa and his collaborators (Iwasa et al. 2006). A stochastic model was also applied to the two-hit model by Frank (2003). In this appendix, we aim to elucidate the relationship between stochastic models and differential equations.

We adopt the notation of Iwasa et al. for the stochastic equations (Iwasa et al. 2006):

A1 $$\begin{aligned} \frac{d f_x}{d t} = r (x-1) f_{x-1} + d (x+1) f_{x+1} - (r+d) x f_x~. \end{aligned}$$

Here, $f_x$ denotes the probability of having *x* cells. The rate *r* represents the cell proliferation rate, and *d* represents the cell death rate. This stochastic model describes the time evolution of the cell number distribution. It consists of a set of coupled equations for $f_x$ with $x=0,1,\ldots ,M$:

A2 $$\begin{aligned} &  \frac{d f_0}{d t} = d f_1~, \nonumber \\ &  \frac{d f_1}{d t} = 2d f_2 - (r+d) f_1~, \nonumber \\ &  \ldots \nonumber \\ &  \frac{d f_{M-1}}{d t} = r (M-2) f_{M-2} + d M f_M - (r+d)(M-1) f_{M-1}~, \nonumber \\ &  \frac{d f_{M}}{d t} = r (M-1) f_{M-1} - d M f_M~. \end{aligned}$$

We can verify that $\frac{d}{dt}(f_0 + f_1 + \ldots + f_M) = 0$. Hence, $\sum _{x=0}^M f_x = \text {const} = 1$, confirming that $f_x$ represents the probability of having *x* cells.

The average number of cells at time *t* is then defined as $N = \sum _{x=0}^M x f_x$. To derive the time evolution of the average cell number *N*, we consider the following set of weighted equations:

A3 $$\begin{aligned} &  0\frac{d f_0}{d t} = 0 \times d f_1~, \nonumber \\ &  \frac{d f_1}{d t} = 2d f_2 - (r+d) f_1~, \nonumber \\ &  2\frac{d f_2}{d t} = 2(r f_1 + 3d f_3 - (r+d) 2 f_2)~, \nonumber \\ &  3\frac{d f_3}{d t} = 3(2r f_2 + 4d f_4 - (r+d) 3 f_3)~, \nonumber \\ &  \ldots \nonumber \\ &  (M-1)\frac{d f_{M-1}}{d t} = (M-1)(r (M-2) f_{M-2} + M d f_M - (r+d)(M-1) f_{M-1})~, \nonumber \\ &  M\frac{d f_M}{d t} = M(r (M-1) f_{M-1} - d M f_M)~. \end{aligned}$$

Summing these equations yields the following:

A4 $$\begin{aligned} \frac{dN}{dt} = (r-d)N~. \end{aligned}$$

If we define the net growth rate as $\lambda = r - d$, we obtain the proliferation equation :

A5 $$\begin{aligned} \frac{dN}{dt} = \lambda N~. \end{aligned}$$

Luria and Delbrück described bacterial growth using this type of differential equation (Lulias and Delbrück 1943). Regarding the distribution of cell numbers, they assumed a Poisson distribution.

Iwasa and his collaborators introduced the transition rate in the following form:

A6 $$\begin{aligned} \frac{dN_t}{dt} = \frac{1}{1-d/r} bN~. \end{aligned}$$

This indicates that the transition rate is proportional to the number of cells.  Thus, we may derive the differential equations for the average number of cells *N* and the number of mutated cells $N_t$ under the assumption $r \gg d$ as

A7 $$\begin{aligned} &  \frac{d N}{dt} = \lambda N - bN~, \nonumber \\ &  \frac{d N_t}{dt} = bN~. \end{aligned}$$

These coupled equations correspond to the Luria-Delbrück distribution (Lulias and Delbrück 1943).  These differential equations were later generalized by Sachs and Hlatky (2010) in the following form :

A8 $$\begin{aligned} \frac{dN_k}{dt} = N_k g_k(N_0,N_1,\ldots ,N_M) + \sum _{j=0}^M N_j Q_{jk},~~~k=0,1,\ldots ,M~. \end{aligned}$$

Here, we adopt the notation used in this paper, modifying the symbols $f_k$ and *N* used by Sachs and Hlatky to $N_k$ and *M*, respectively. Thus, the equations of Sachs and Hlatky represent differential equations for the average numbers of cells, where the index *k* denotes different stages of cellular mutation. In our case, we set $M=2$: $k=0$ corresponds to the normal cell $Rb^{+/+}$, $k=1$ to the singly mutated cell $Rb^{+/-}$, and $k=2$ to the doubly mutated cell $Rb^{-/-}$.

To formulate our differential equations (Eqs. 1, 2, 3), we consider that the total number of retinal cells is fixed at $N_m$, and that the mutated cells also proliferate. Thus, we model the function $f_k(N_0,N_1,\ldots ,N_M)$ introduced by Sachs and Hlatky as

A9 $$\begin{aligned} f_k(N_0,N_1,N_2) = 1 - \frac{N_0 + N_1 + N_2}{N_m}~. \end{aligned}$$

The resulting differential equations are represented schematically in Fig. 2 in the main text.

## Appendix B: Two-hit Model

The multi-hit theory proposed by Armitage and Doll was developed to describe cancer incidence rates (Armitage and Doll 1954). This theory does not establish a direct connection with the mutation processes at the cellular level. Consequently, when cancer incidence rates begin to rise around the age of 60, it is generally assumed–though somewhat vaguely–that multiple successive mutations must have occurred at the cellular level. In contrast, in the case of retinoblastoma (Rb), only two hits to the Rb gene in a retinal cell are required, and the mutations, the source of the hits, occur even before birth. In this context, it is difficult to directly associate the Rb case with typical somatic mutation mechanisms. Since the two-hit model was employed in Knudson (1971), we describe it here to highlight the differences from the 2P2H model. The two-hit model was discussed from a different perspective by Frank recently (Frank 2005). In this framework, time is assumed to begin at birth, since comparison is made with the clinical incidence rate.

We formulate the equations of the two-hit model for clinical incidence rates. For bilateral Rb in the hereditary group, only one additional hit is required for cancer development, with the hit rate denoted by *k* to distinguish it from the mutation rate *a*.

A10 $$\begin{aligned} &  \frac{d N_1}{dt}= - kN_1~, \nonumber \\ &  \frac{d N_2}{dt}=k N_1~. \end{aligned}$$

The solutions to the above differential equations are:

A11 $$\begin{aligned} &  N_1=\bar{N}_1 e^{-kt}~, \nonumber \\ &  N_2=\bar{N}_1 (1-e^{-kt})~. \end{aligned}$$

Here, $\bar{N}_1$ denotes the number of $Rb^{+/-}$ cells that are converted to $Rb^{-/-}$ cells through the hit rate *k*. The survival rate $S_B$ and the incidence rate $I_B$ are given by:

A12 $$\begin{aligned} &  S_B=(\bar{N}_1-N_2)/\bar{N}_1 = e^{-kt}~, \nonumber \\ &  I_B=\frac{d N_2}{dt} = k \bar{N}_1 e^{-kt}~. \end{aligned}$$

Note that calculation of the incidence rate $I_B$ requires knowledge of $\bar{N}_1$, which represents the total number of potential incidence cases.

For unilateral Rb in the non-hereditary group, two hits are required for cancer incidence. Accordingly, the differential equations are:

A13 $$\begin{aligned} &  \frac{d N_0}{dt}= -2kN_0 \end{aligned}$$

A14 $$\begin{aligned} &  \frac{d N_1}{dt}= 2kN_0 - kN_1 \end{aligned}$$

A15 $$\begin{aligned} &  \frac{d N_2}{dt}= kN_1 \end{aligned}$$

Here, the factor of 2 in the equations accounts for the two possibilities, $Rb^{+/-}$ and $Rb^{-/+}$, both leading to the final state $Rb^{-/-}$. The solutions to these differential equations are:

A16 $$\begin{aligned} &  N_0=\bar{N}_0 e^{-2kt} \end{aligned}$$

A17 $$\begin{aligned} &  N_1=2\bar{N}_0 (e^{-kt} - e^{-2kt}) \end{aligned}$$

A18 $$\begin{aligned} &  N_2=\bar{N}_0 (1 + e^{-2kt} - 2e^{-kt}) \end{aligned}$$

Here, $\bar{N}_0$ represents the total number of $R^{+/+}$ cells that are converted to $R^{-/-}$ through two hits at the rate *k*. The survival rate $S_U$ and the incidence rate $I_U$ are given by:

A19 $$\begin{aligned} &  S_U=(\bar{N}_0 - N_2)/\bar{N}_0 = 2e^{-kt} - e^{-2kt} \end{aligned}$$

A20 $$\begin{aligned} &  I_U=\frac{d N_2}{dt} = 2\bar{N}_0 k (e^{-kt} - e^{-2kt}) \end{aligned}$$

The survival rate can be written approximately as

A21 $$\begin{aligned} S_U\sim 1-k^2t^2 \end{aligned}$$

We set the initial value at $\bar{N}_1=2.4\times 10^{-5}$ based on the observed bilateral clinical incidence. We then determine the hit rate as $k_B = 0.112/\text {month}$ with a 95% confidence interval (0.095, 0.131) using the maximum likelihood method. The results are shown in Fig. 9, where good agreement is observed between the two-hit model and the clinical 168 bilateral data (out of 378 data) points of Mastrangelo et al. (2008).

For the unilateral case, we set the initial value at $\bar{N}_0=3.4\times 10^{-5}$ based on the observed unilateral incidence. We note here that the use of the initial value of $\bar{N}_0=1$ does not provide the proper magnitude of the incidence rate. Similarly, we determine the hit rate as $k_U=0.071/\text {month}$ with a 95% confidence interval (0.064, 0.078) using the maximum likelihood method. The results are also shown in Fig. 9, and good agreement on the age profile is again observed. Thus, in fitting the incidence rates for both bilateral and unilateral cases, the two-hit model reproduces the observed profiles (Mastrangelo et al. 2008), as initially noted by Knudson using survival function analysis with 48 data points (Knudson 1971). We emphasize here again that the magnitude of the unilateral cases is not reproduced by the two-hit model. Additionally, the hit rates $k_B$ and $k_U$ differ each other, while close to the duration of the latent process $\mu ^{-1}=1/11.8\text {month}=0.085/\text {month}$. The time scale of $k_B, k_U$ is completely different from the mutation rate $a=6.8\times 10^{-9}/$day.

We emphasize again that the multi-hit theory of Armitage and Doll was intended to reproduce cancer incidence rates without considering latent periods and the mutation rates. Therefore, it remains difficult to directly relate the parameters of the hit theory to mutation rates at the cellular level.

## Appendix C: Some Trial of Second Mutation after the Birth on the Unilateral Rb

For the U cases, we show that their magnitude and age profile cannot be reproduced if we only consider mutations during the growth phase, as shown in Fig. 6. Here, we used the same latent function to estimate the absolute number of the U cases along with the age profile of Rb clinical cases. By taking the same mutation rate $a=6.8\times 10^{-9}$/day without proliferation, the calculated results are shown by the red curve in Fig. 10, where the incidence profile starts to grow gradually due to the latent effect and overtake the observed age profile around $T=40$ months. Adding the contributions from the G period, we obtain the black curve, where the calculated results are smaller than the incidence rates in the range of 15 to 30 months, and larger than those after 40 months.

## Declarations

### Data Usage

We used data published in literatures, which are cited in this article.
